# Smart Swarms of Bacteria-Inspired Agents with Performance Adaptable Interactions

**DOI:** 10.1371/journal.pcbi.1002177

**Published:** 2011-09-29

**Authors:** Adi Shklarsh, Gil Ariel, Elad Schneidman, Eshel Ben-Jacob

**Affiliations:** 1 School of Computer Science, Tel Aviv University, Tel Aviv, Israel; 2 School of Physics and Astronomy, Tel Aviv University, Tel Aviv, Israel; 3 Department of Computer Science and Applied Mathematics, Weizmann Institute of Science, Rehovot, Israel; 4 Department of Mathematics, Bar Ilan University, Ramat Gan, Israel; 5 Department of Neurobiology, Weizmann Institute of Science, Rehovot, Israel; 6 The Center for Theoretical and Biological Physics, University of California San Diego, La Jolla, California, United States of America; Princeton University, United States of America

## Abstract

Collective navigation and swarming have been studied in animal groups, such as fish schools, bird flocks, bacteria, and slime molds. Computer modeling has shown that collective behavior of simple agents can result from simple interactions between the agents, which include short range repulsion, intermediate range alignment, and long range attraction. Here we study collective navigation of bacteria-inspired smart agents in complex terrains, with adaptive interactions that depend on performance. More specifically, each agent adjusts its interactions with the other agents according to its local environment – by decreasing the peers' influence while navigating in a beneficial direction, and increasing it otherwise. We show that inclusion of such performance dependent adaptable interactions significantly improves the collective swarming performance, leading to highly efficient navigation, especially in complex terrains. Notably, to afford such adaptable interactions, each modeled agent requires only simple computational capabilities with short-term memory, which can easily be implemented in simple swarming robots.

## Author Summary

Many groups of organisms, from colonies of bacteria and social insects through schools of fish and flocks of birds to herds of mammals exhibit advanced collective navigation. Identifying the minimal features of biologically-inspired interacting agents that can lead to emergence of “intelligent” like collective navigation and decision making is fundamental to our understanding of collective behavior, and is of great interest in artificial intelligence and robotics. Previous models of collective behavior of agents, which relied on static interactions of repulsion, orientation (alignment), and attraction, have shown the emergence of collective swarming. Here we show the advantage of performance adaptable interactions for navigation of groups in complex terrains. Each agent senses the local environment and is then allowed to adjust its interactions with the other agents according to its local environment – by decreasing the peers' influence while navigating in a beneficial direction and vice versa. We found that inclusion of such adaptable interactions dramatically improves the collective swarming performance leading to highly efficient navigation especially in very complex terrains.

## Introduction

Many organisms exhibit complex group behavior [1]–[10], including collective navigation observed in the flight of birds [11], trail organization in ants [12], and swarming of locust [13], fish [14] and bacteria [15], among others. The aggregation results in highly complex collective behavior, with new functionality and computational ability. Simple interaction models, which describe how each agent acts according to the result of a ‘computation’ it performs on the locations of the other agents, have been used to demonstrate and study the fundamental building blocks of complex group behavior [16]–[24].

In computational models, swarming behavior can arise from simple rules, and in particular demonstrate qualitive (and sometimes quantitative) features of collective behavior observed in nature: Vicsek et al. [16] introduced the ‘self-propelling particles’ (SPP) model, in which the motion of each individual is determined by the mean orientation of its local neighborhood with some noise induced perturbation. The SPP model can exhibit random or coherent motion of group clusters depending on the particle density and on the noise of each individual; high density and low noise results in a coherent group motion. Later derivatives of the SPP model included individual preferential movement directions, collision avoidance, and attraction [17]–[23]. Couzin et al. [17], [18] studied a model in which the direction of motion of each individual is determined by a set of rules: repulsion (from neighbors who are too close), attraction (to prevent fragmentation), alignment (of velocity directions and speed), and reaction to the environment. A swarm using these interaction rules can come to a collective ‘decision’ about its direction of movement without leadership and a small fraction of individuals ‘in agreement’ are needed for such a cohesive decision to be made. Recently, Torney et al. [25] presented a model in which the individual agents adapt their interactions according to local conditions. A special feature of this model is that it leads to the emergence of collective navigation although each agent does not possess individual navigation capabilities.

Even bacteria show remarkably sophisticated collective behaviors. Some bacteria strains can form large colonies with intricate complex architectures, which allows them to expand efficiently by taking advantage of the available resources [26]–[29]. They construct intricate multicellular structures utilized for protection and cooperation of cells [30]–[33]. In addition, bacteria display complicated movement dynamics, in which cells organize into vortices, form traffic lanes, or move collectively in a common direction [34]–[36]. Bacteria swarming behavior in colonies was explained by considering attractive and repulsive forces between colony parts [10], [28], [37], [38], communication capabilites [39]–[43], physical interactions between cells, and forces from the environment [44].

Bacteria navigate using chemotaxis, i.e., moving according to gradients in the chemical concentration [45]–[49]. Bacteria are too small to detect the chemical gradients across their body receptors, and thus detect the concentration as they swim, and delay their tumble if the concentration increases. As a result, they make longer excursions towards areas of higher concentration. Each bacterium may only acquire local and partial cues from the environment, but as a group bacteria can navigate through challenging environments. In such cases, the optimal local direction may be completely independent of the global environment. In addition, microorganisms are especially sensitive to noise, due to stochastic variations in their internal mechanisms, sensory system, and the external environment. Information pooling was shown to improve decision making in animal groups [1], [50]–[52], such as the accuracy of navigating birds. In addition, it has been shown that schooling can improve the collective ability of groups of chemotactic organisms, such as bacteria, to climb gradients [53].

Interaction between individuals such as repulsion, alignment, and attraction, may exist in bacteria due to the associations between single cells by mechanical and chemical means. Mechanical interactions can result in collision or adhesion of cells. Chemical interactions, by secretion and detection of various diffusible chemicals, can result in repulsion or attraction. In high densities, interactions between elongated cells cause alignment of cell bodies and velocities.

Here, motivated by bacteria swarming, we study the collective behavior of agents with self-navigation capabilities (particularly, a tractable variant of chemotaxis) and performance dependent adaptable interactions. Specifically, when the change of chemical concentration is positive, an agent is more likely to continue in its previous direction, thus, decreasing the influence of the other agents, and vice versa. This implies that the interaction network among agents is plastic – similar in spirit to the approach in machine learning and neuroscience [54]–[56] and the recent work by Torney et al. [25].

The current approach enables quanitative comparison between the efficiency of collective navigation in the case of static and adaptable interactions. We found that the adaptable interactions become more important for more complex terrains. We also found that collective navigation of agents with adaptable interactions is more robust to the initial conditions, to the internal noise in the system, and to the values of the interactions.

## Methods

### Modeling the terrain

**Figure 1:**
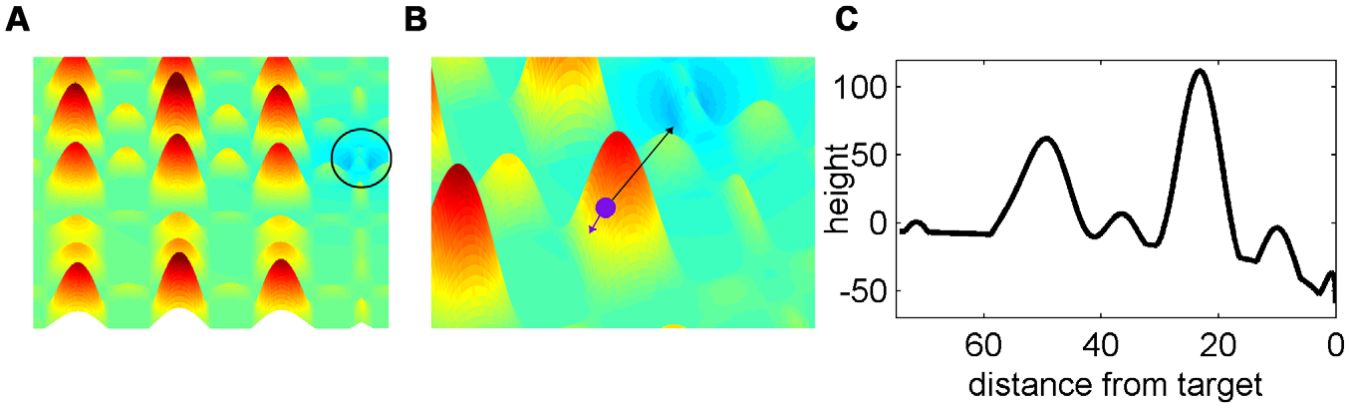
Illustration of the terrain representing the chemical concentration map. A. Overview of the terrain given by 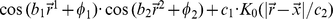. B. Illustration of a single agent and the global direction (in black) to the target compared to the local gradient (in purple) of the concentration map. C. Illustration of the terrain height along a straight trajectory from the swarm's starting position to the target.

We studied the navigation efficiency of swarms in a complex two-dimensional terrain with obstacles. The structure of the terrain was given by a static concentration map (Figure 1A) of the form:
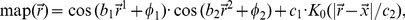
(1)where 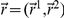, 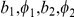 are constants defining the periodic variations of the terrain, and 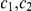 are constants relating to the underlying chemical diffusion gradient. 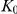 is the modified Bessel function of the second kind (which is the solution to the differential equations related to diffusion in two dimensions) and 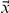 is the location of the target. The ‘topography’ was one of mountains and valleys, representing locally changing chemical concentration that is ‘overlaid’ on a global concentration valley. In particular, the modeled terrain had local minima and bottlenecks. The mountains represent low chemical concentration whereas the valleys represent high concentration. The target was the lowest point on the map – which was the maximum concentration point (Figure 1B,C). The concentration map was chosen as a simplified model of a problem with local variations and local minima (see Text S1, Figure S1), motivated by real navigational problems in bacteria swarms. Agents that are only capable of local measurements are thus faced with an extremely challenging navigation task.

### Movement of individual agents

Three main factors influence the movement of the agents. The first is internal noise of each agent, the second is its environment, and the third is interaction with other agents.

On each time step, the location 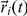 and the direction of the velocity 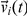 of agent 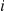 were updated according to the following rules:
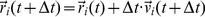
(2)

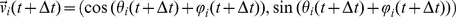
(3)where 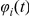 represents internal noise taken from a Gaussian distribution with variance 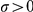, 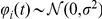, and 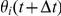 is an agent's new angle (see below).

**Figure 2:**
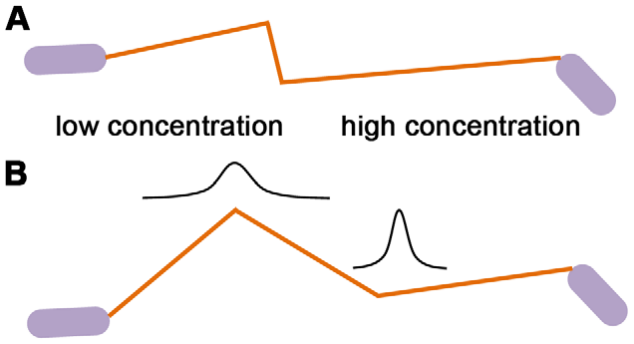
Illustration of the motion of an individual bacterium and an individual agent. A. A bacterium's motion in chemical gradients is a biased random walk towards areas of higher chemical concentration. A bacterium makes longer excursions when it moves in areas of higher chemical gradients after which it performs a random switch of direction, known as a tumble. B. The motion of an individual agent is composed of equal sized excursions after which the agent tumbles. The tumble is a change in direction taken from a Gaussian distribution whose variance is a function of the chemical concentration. When the chemical gradient is small, the variance is large, and vice versa.

The movement of an individual agent devised here was inspired by chemotaxis in swimming bacteria. A bacterium follows chemical gradients by decreasing its tumbling frequency in high gradients, whereas the tumbling angle is random (Figure 2A). In the model we implemented this mechanism by maintaining an equal tumbling frequency whereas the tumbling angle depended on the chemical gradient (Figure 2B). We note that both approaches produce the same effect statistically. An agent moves forward and after a time step 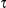, it changes direction by an angle, 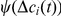, which is randomly chosen from a Gaussian distribution, 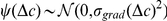. The distribution variance was given by
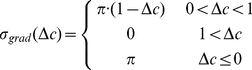
(4)where 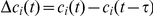 is the chemical gradient and 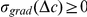. We note that when the speed is constant, the agent will move forward by 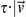; since in the model 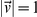, the forward motion length equals 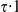. The variance 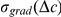 decreases with an increase in the chemical gradient, biasing the direction of motion up chemical gradients.

During a forward motion, an agent's new angle 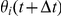 was the angle of its previous velocity direction, 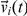, and during a tumble, the new angle was given by
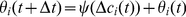
(5)


### Interactions between the agents

#### Two-agents interaction

**Figure 3:**
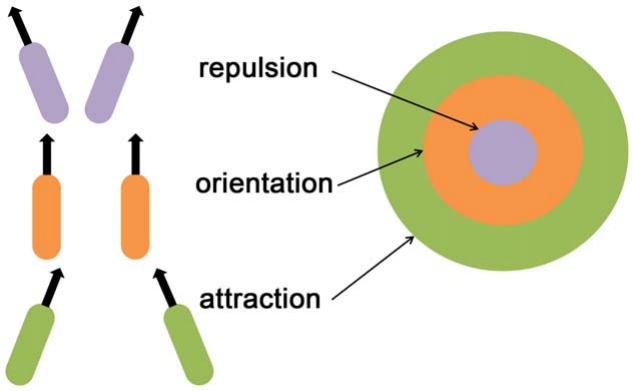
Illustration of agent-agent interactions in the swarm model. Agents repel from close agents, align with intermediate agents, and move towards far agents.

An individual agent 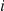 will repel from another agent 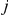 when it is in the range 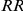; it will align its velocity with the direction of the other agent when it is in the range 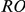, and it will go towards it if it is in the range 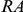 (Figure 3).

#### Many-agents interaction

We denote 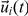 to be the direction resulting from the group interactions of agent 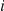, if there is more than one agent in any of the interaction ranges. If there are any agents within distance 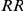, it will try to avoid collision, and will thus set its velocity to be:
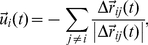
(6)where 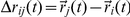, and the sum is over all 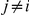 such that 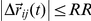. If there are no agents in the range of repulsion, agent 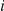 will align with agents within distance 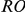 and move towards agents within distance 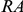 according to: 
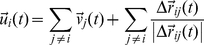
(7)where for the left term, the sum is over all 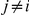 such that 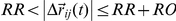, for the right term, the sum is over all 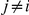 such that 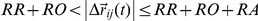, and 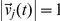.

### Combination of chemotactic motion with group interactions

During a forward motion, an agent's new angle 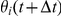, was selected by:
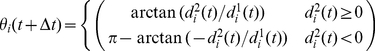
(8)where the direction 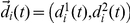 was a combination of an agent's previous velocity direction and the group interactions and was defined as
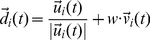
(9)where 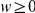. During a tumble, the new angle was selected as in equation (5).

### Adaptable interaction model

In the model presented above, the weight 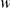, which determines the balance between group interactions and individual direction, was fixed (see equation (9)). We now consider the case of an adaptable 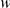 that is adjusted according to local conditions. Specifically, we decreased the weight of agent 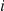, 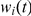, when the agent moved in the direction of decreasing gradient and vice versa. Among many possible ways to control the weight that would accomplish this goal we used a simple update scheme where 
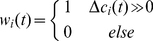
(10)


The new direction was then given by: 
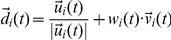
. Note that for 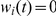, the new direction depends only on group interactions. In contrast, 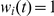 corresponds to equal balance between group interactions and individual direction.

### Group movement characteristics

The path length, which is equal to the navigation time multiplied by 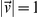, was computed as the median path length, and corresponds to the time it took half of the agents in the group to reach the target. The group alignment was given by
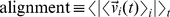
(11)


The group alignment is the average alignment between individuals over the trajectory. Notice that the group alignment is in the range 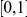. The group's cohesion was assessed by the number of clusters at the end of the simulation where clusters were separated according to the region of interactions between agents bounded by 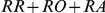.

### Selection of the model parameters

In nature, an organism's motion mechanism or interaction range should fit the common characteristics of its environment. The challenge in choosing the model parameters is to select them to fit the terrain, since for each terrain there is a different set of parameters that is most efficient. We fixed the terrain and chose the model parameters, including the time step in which an agent moves forward before it tumbles, 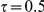, and the group interaction radii, 
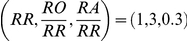
. The terrain characteristic sizes include the terrain characteristic length, which corresponds to the typical distance between two adjacent peaks or valleys, and the characteristic slope of the mountains or valleys. We found 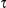 to have a strong effect on the behavior of individual agents. When the length of an agent's forward motion was small compared to the terrain's characteristic gradient, the agents were unable to detect changes in the concentration, causing their motion to become more random. When the forward motion ‘runs’ were large compared to the terrain's characteristic length, then agents could not follow the smooth terrain's gradient since their motion was made of large independent leaps in an irregular terrain. Moreover, small radii of repulsion compared to the terrain's characteristic gradient, resulted in groups aggregating together and thus measuring more similar concentrations, which caused the groups to get stuck in local minima. In general, increasing the characteristic gradient of the terrain (corresponding to a ‘heightening’ of the mountains and a ‘deepening’ of the valleys) makes the task harder since agents are more likely to settle in local minima.

## Results

We simulated different groups of 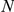 moving agents navigating on a complex terrain, given by equation (1), with 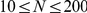. The initial conditions were set such that the agents' locations, 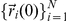, were uniformly distributed around the starting position inside a circle with radius 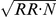, which meant that the group was not fragmented and all the agents were interacting with other agents. The agents' velocities were 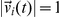, and their directions were uniformly distributed over all directions, 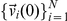.

### Comparison of typical realizations of the models

**Figure 4:**
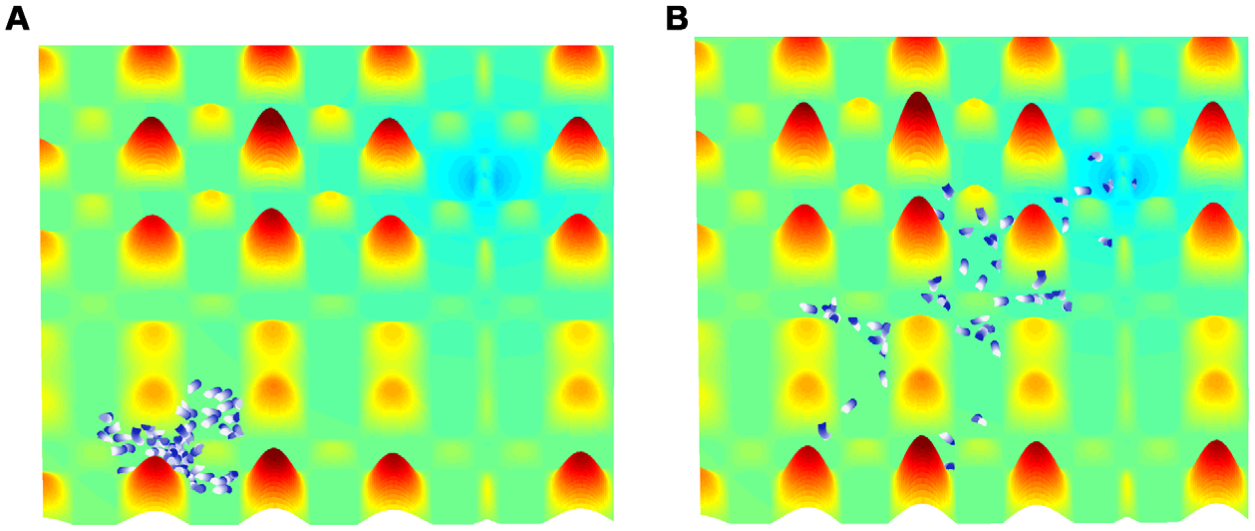
Frames from the simulation show independent agents moving towards the target. A. Simulation step 100. B. Simulation step 2000. 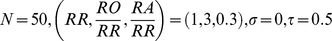.

**Figure 5:**
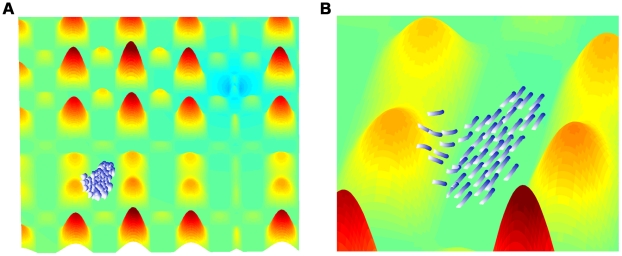
Frames from a simulation of interacting agents moving collectively towards the target. A. Simulation step 200 from starting position. B. Zoom in on the group in the left frame. Simulation parameters are as in Figure 4, 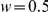.

**Figure 6:**
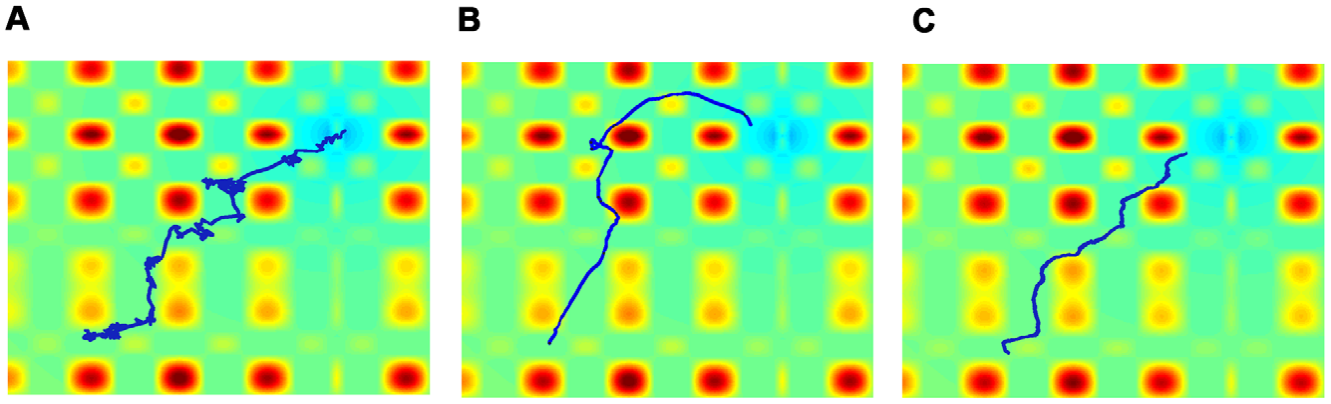
Examples of individual paths of agents using the different interaction mechanisms. A. The path of a single independent agent shows a biased random walk towards the target. B. The path of a single interacting agent shows directed movement towards the target with global errors. C. The path of a single agent with adaptable interactions shows directed movement towards the target with smaller global errors. Simulation parameters are as in Figure 5.

**Figure 7:**
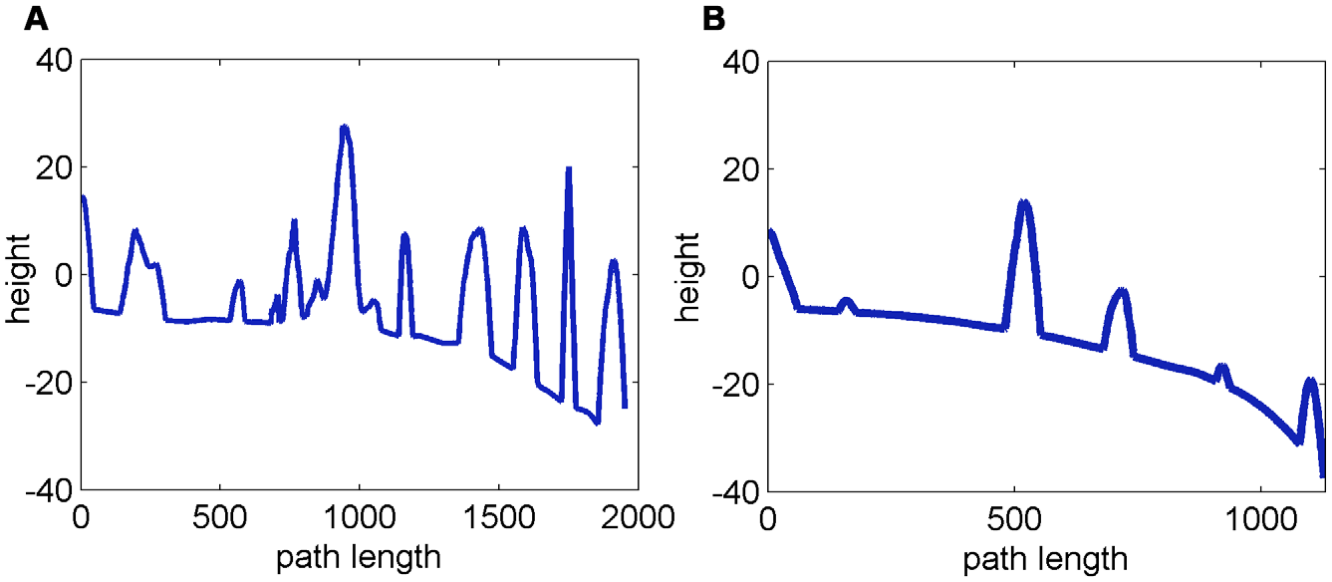
Height along the path of the group center. A. For interacting agents. B. For agents with adaptable interactions. Simulation parameters are as in Figure 5.

Figure 4 shows the typical results for the movement patterns of a group of 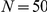 independent agents on a complex terrain. An example of a similar group of interacting agents, with static interactions is shown in Figure 5. Examples of the paths of a single agent under the independent model, the static interactions model, and the adaptable interactions model are shown in Figure 6. These snapshots are reproduced from full simulation runs (see full movies in Supplementary Information Video S1-[Supplementary-material pcbi.1002177.s006] 3). The height along the navigation path of the group center for interacting agents and agents with adaptable interactions are shown in Figure 7. The groups demonstrated extremely diverse movement patterns: groups split and collide, they bump into the mountains, single agents break away from the main group, groups move away from the target, or circle around in one place (See Video S4–[Supplementary-material pcbi.1002177.s009] 6).

### Navigation of independent agents and interacting agents

A single agent performing an independent search on the concentration map would move in a biased random walk fashion towards the target with a profuse amount of local ‘errors’ with respect to the global target. A group of agents navigating independently would converge as a group towards the target, with each agent performing its own biased Brownian motion. We quantify the error fraction by:
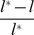
(12)where 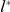 is the median path length (the time it took half of the elements in the group to reach the target multiplied by 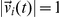) and 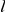 is the distance between the starting position and the target, and found that the error fraction was approximately 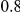. We chose to measure the median path length and not the average, to prevent the bias of rare instances of extremely long path lengths.

In complex terrains, interacting agents were less sensitive to local noise due to the group's influence, and the group as a whole was cohesive and aligned; this is in agreement to what has been suggested by Grunbaum [53]. The average error fraction (equation (12)) in this case was approximately 0.65, considerably less than the independent agents. Although the interacting agents were more robust to local noise than the independent agents, they still had a large error fraction.

### The effect of the balance between the group's influence and individual direction on interacting agents

**Figure 8:**
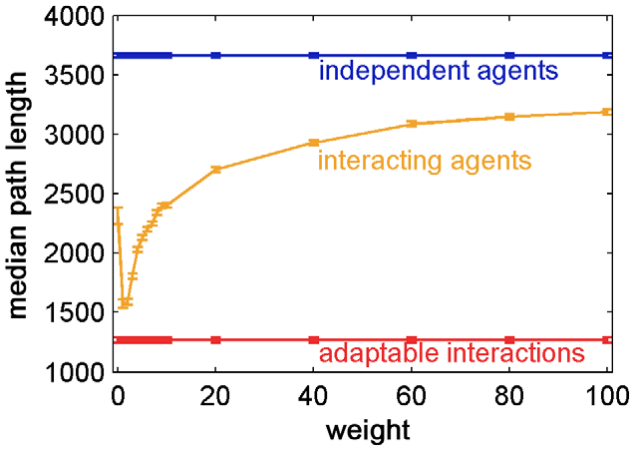
The path length of interacting agents is optimal in an intermediate range of weights. The median path length is shown as a function of the constant fixed weight of interacting agents. The average median path length of independent agents and agents with adaptable interactions is shown for comparison. Simulation parameters are as in Figure 5, 100 rounds. Error bars represent standard error.

The value of the weight 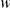, which balances the effect of the individual direction of motion (based on local information) and the group's influence (equation (9)), had an immense effect on the group's behavior and organization (Figure 8). Large weights imply that the agents were mostly influenced by their own direction of motion and that the interactions between them were weak, which means that the group would behave more like independent agents. Small 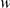 imply that agents were strongly influenced by their neighbors, which results in excessive conformity. This could also mean that agents would be led off the track by small errors and perturbations that would be amplified by the positive feedback in the group. There is an intermediate range of weight values that lead to optimal median path lengths, shown in Figure 8.

### The effect of adaptable interactions

The adaptable interaction rules (equation (10)) kept the group cohesive and aligned as in the case of static interactions, but, we found that such groups were not sensitive to local noise, and also less sensitive to global noise. The error fraction (equation (12)) in this case was approximately 0.5 – considerably less than both the independent and the fixed interaction agents. The adaptable interactions affect the error in the global group movement by modulating the positive feedback in the group's self-influence with feedback from the environment.

**Figure 9:**
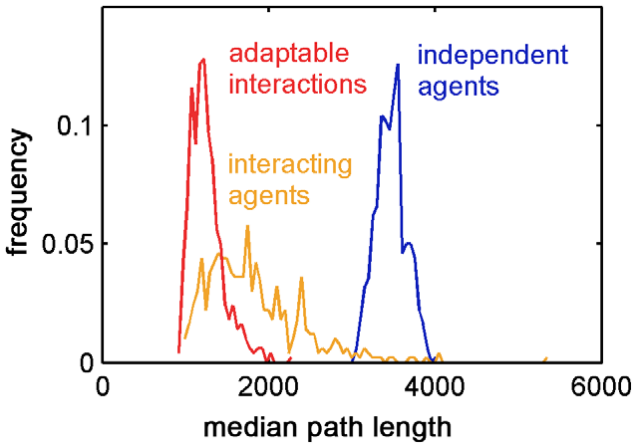
Distribution of navigation path lengths for the different interaction mechanisms. The mean median path length of agents with adaptable interactions is lower than that of both interacting and independent agents. The mean path length of independent agents is significantly larger. The variance of the path lengths of interacting agents is larger than that of the other mechanisms due to positive feedback. Simulation parameters are as in Figure 5, 500 rounds.

The median length of the path of agents with adaptable interactions was smaller than that of the fixed interacting agents – even for optimal weight values (shown in Figure 8) – which was smaller than that of the independent agents (Figure 9). Interestingly, the variance of the distribution of the path length of the interacting agents was higher than in the other mechanisms. We suggest that this is due to the positive feedback, which may sometimes lead groups off track in the presence of a global noise source. As illustrated in Figure 9, the variance of the distribution of agents with adaptable interactions was smaller than the variance of interacting agents, giving the adaptable mechanism yet another advantage – having the group find the target quickly with certainty.

**Figure 10:**
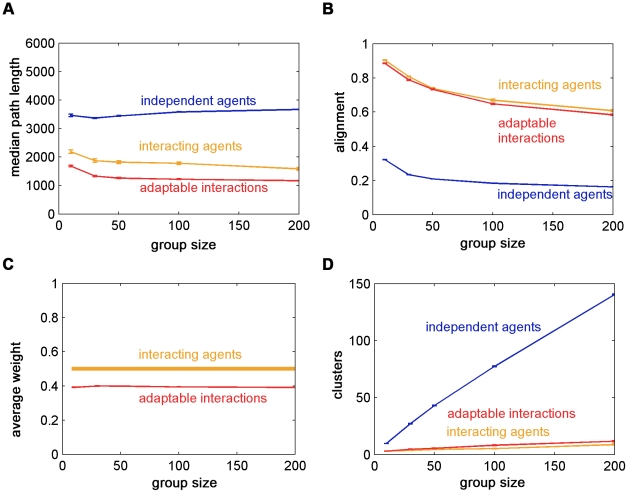
Comparison of the interaction mechanisms as a function of group size. A. Agents with adaptable interactions find the target faster than interacting agents. Both are much faster than independent agents. The median path length increases as a function of group size for independent agents due to an increase in the sample size. It decreases for interacting agents and for agents with adaptable interactions due to the collection of information from more agents. B. Alignment decreases as a function of group size. Independent agents are not aligned while agents with adaptable interactions are less aligned than interacting agents. C. For agents with adaptable interactions, group average weight is not strongly dependent on group size. The average weight of agents with adaptable interactions is lower than the constant predetermined weight of interacting agents. Nonlinearity cause lower average weights, which correspond to higher conformism and implies higher group alignment, to couple with lower alignment. D. The average number of clusters increases with the group size. Independent agents are highly clustered while agents with adaptable interactions are more clustered than interacting agents. Simulation parameters are as in Figure 8, apart from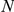. Error bars represent standard error.

We compared the group's movement characteristics as a function of group size for the three interaction mechanisms. Figure 10A shows that agents with adaptable interactions found the source faster than the groups using the other mechanisms. The navigation time increased as a function of the group size for independent agents, due to convergence to the true average navigation time, since there is a larger probability for rare occurrences with an increase in the number of agents. However, the path length decreased as a function of group size for interacting agents and agents with adaptable interactions, due to collection of information from more agents. Alignment decreased with group size and, as expected, independent agents had very low alignment (Figure 10B). For agents with adaptable interactions, we found that the alignment and the average weight (which equals the proportion of agents with 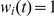), are lower than that of fixed interactions agents (Figure 10C). This may seem contradictory since lower weights are expected to increase the coordination in the group, which leads to higher alignment. We note however that for each agent 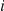 in our case, 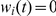 or 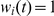, whereas for the fixed interaction agents, 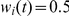 for all agents at all times. This amounts to a non-linear effect of the weights on the alignment. The average number of clusters was the highest for independent agents, having no interactions. The number of clusters of agents with adaptable interactions was slightly higher than that of the interacting agents due to the nonconformity (Figure 10D).

### The effect of noise on group behavior

Our model has three sources of stochasticity: The first one is external, imposed by the surface variations (equation (1)). The second is the Gaussian noise related to the measured concentration gradient, applied by switching direction (equation (4)). The third is the internal noise related to the selection of the direction (equation (3)).

**Figure 11:**
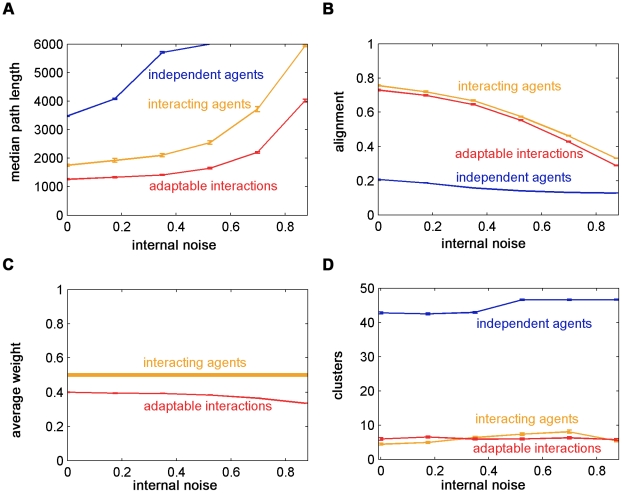
The effect of internal noise on group behavior. A. Diversity in the agents' movements resulting from internal noise causes an increase of median path lengths among all the mechanisms. The effect on the independent agents is the most devastating and groups did not complete the task by the end of the simulation. The effect on the interacting agents is larger than the effect on agents with adaptable interactions. B. Group alignment decreases with 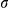. For independent agents, alignment decreases due to the individual loss of biased motion towards the target because of the noise. C. The average weight of agents with adaptable interactions, which is lower than the constant predefined weight of the interacting agents, reduces further with 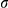, implying groups become more conformist. D. Clustering is weakly affected by 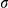. Independent agents are more clustered due to loss of biased motion. Interacting agents become slightly more clustered with 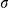. Agents with adaptable interactions are generally slightly more clustered than interacting agents but their clustering is not affected by 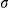. Simulation parameters are as in Figure 8, apart from 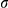. Error bars represent standard error.

To assess the effect of individual agent stochasticity on the group collective navigation, we deflected each agent's movement direction by Gaussian noise with a constant variance, 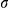. We found that the path length of the group increased with 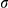, while the manner by which it increased was dependent on the interaction mechanism (Figure 11A). Independent agents were most vulnerable to this noise and for high values they failed to complete the task. Agents with fixed interactions and agents with adaptable interactions were affected similarly by the noise, although interacting agents were affected more strongly. We found that alignment decreased as a function of 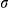 (Figure 11B). The alignment of independent agents also decreased since the noise disrupted the independent biased random walk to the target. In addition, we found that the average weight of agents with adaptable interactions decreased as a function of 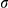 (Figure 11C). Similar to the effect of adaptable interactions on the average weight and alignment, we found that lower average weights implied low alignment. Unlike the performance of agents with adaptable interactions, here the noise causing this led to longer navigation paths.

**Figure 12:**
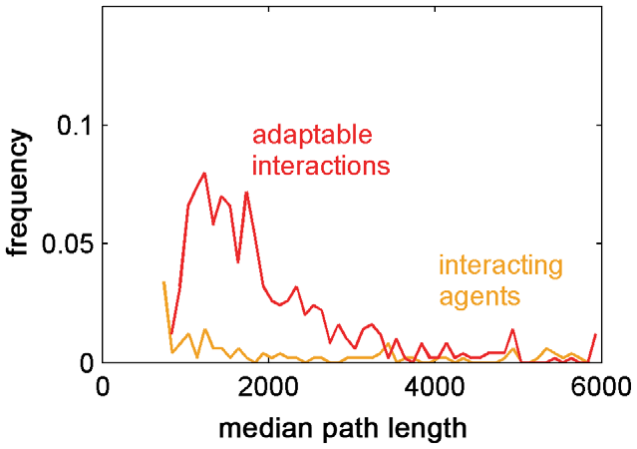
Distribution of median path lengths with diverse forward motion runs. When the sizes of the agents' forward motion runs, denoted by 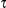, are diverse, the mean median path length of agents with adaptable interactions is much lower than the other mechanisms, which are not clearly visible in the graph since they exceed the simulation length. Simulation parameters are as in Figure 9, apart from the previously constant forward motion run; here 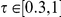.

Next, we let each agent pick a forward run time, 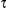, taken from a uniform distribution in the range 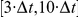 of values that fit the terrain. We then asked how this diversity affected the path lengths of the groups under the three interaction mechanisms. The distributions of the median path lengths reflect that adaptable interactions resulted in considerably shorter paths than that of independent and interacting agents, for which the path lengths were longer than the simulation length (Figure 12).

### The effect of the radii of interactions

The interactions between the agents – repulsion, alignment, or attraction – depend on the distance between them as defined by the radii of interactions, 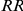, 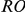, and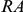, respectively; these radii of interactions determine the collective behavior, in particular, alignment and task performance [18]. We found performance dependent adaptable interactions to be more robust to the values of the radii of interactions than the other mechanisms (Figure S2, Figure S3). See more details in the Text S1.

## Discussion

We introduced a collective behavior model of a group of interacting agents, in which each group member senses the environment and adaptively weighs its own evidence and the behavior of its neighbors to navigate in a complex environment.

We expanded a model that originated from the self propelling particles model and has been used to describe swarming in many complex systems [17]–[23], [25], [53], [57]. We investigated the navigation capabilities of the swarm in a complex terrain and showed that independent agents create fragmented groups while each agent performs an independent biased random walk towards the target. Interacting agents were far better in finding the target than independent agents, and also demonstrated emergent collective swarming, but were affected strongly by global noise due to positive feedback. Previously, Torney et al. [25] showed that adaptable interactions can lead to the emergence of collective navigation in swarms composed of agents that do not posses navigation capabilities as individuals. Here, we studied collective navigation of agents which do possess navigation capabilities as individuals while focusing on the advantage of performance dependent interactions.

When we added a learning mechanism to the network of agent-agent interactions, these swarms had a higher probability of finding the source, and significantly faster. Moreover, performance-dependent adaptive interactions improved the efficiency of the collective navigation beyond that of agents with static interactions, even for an optimal set of static interaction parameters. The adaptable interactions enabled agents to adjust the weight they gave to their neighbors according to local conditions. We used a hard limit weighting, in which agents either followed their neighbors or balanced equally between them and their individual direction, and this was enough to significantly improve the navigation efficiency of swarms in a complex terrain. We note that we did not add memory beyond the measurement of the change in concentration, which already exists in the navigation of an independent agent, or additional computational capabilities to the agents. Using the immediate environment as a teacher the weights of each agent in the network changes dynamically. This gave a form of noise reduction, where the influence of erroneous agents on the system was reduced, and the power of sub-groups changed and resulted in a dynamically shifting leading cluster of agents that comprises of only the most successful performers.

We found that the adaptable interactions model is more robust to internal noise and to diversity in the agents' control mechanism parameters. In particular, the model was robust to the radii of interactions. The system of agents with adaptable interactions changed dynamically according to each agent's success and as a result, the system as a whole transforms into a robust yet “plastic” network.

Models of swarm intelligence and their analysis have the potential to export ideas and algorithms from nature into novel computational tools, including distributed algorithms for optimization and other complex problems in addition to mechanisms for robotic systems [58]-[61]. The model we studied here can be viewed as a distributed network of sensors, with the capability of having local effects on each other. The problem at hand is a function optimization task, where the function samples contain local and global errors. Each sensor can only sample the function at one position and the next sampling position is in the local proximity of the previous one. We investigated how the local effects or interactions between the sensors affect the function optimization time of the network under different conditions. We found that adaptable interactions benefit the system as a whole in a complex navigation task making it faster to find the target under more diverse conditions than before.

In the current study, our swarms constituted of identical individuals with equal measurement capabilities. Natural extension would be investigating the effect of variability, for example in the interaction ranges and noise distributions of agents, on the swarm's collective navigation performance. It is known that many biological mechanisms benefit from variability in the system in the presence of noise [62]-[64]. We expect agent variability to be advantageous for navigation in the case of both spatial and temporal noise. The combination of sensor diversity and adaptable interactions can constitute a solution to navigation in the presence of spatial and temporal noise such as in the case of a time-changing terrain.

Bacteria have developed various communication capabilities such as direct and indirect cell-cell physical and chemical interactions, chemical signaling, such as quorum sensing, and chemotaxis signaling [30], [32], [36], [40], [43], [65]–[67]. Thus, the communication mechanisms necessary to sustain adaptable interactions already exist in bacteria; in fact, the interaction capabilities found in some strains of social bacteria are far more sophisticated and have yet to be understood [68]. Adaptable interactions, similar to what we have suggested, may be found in other groups of simple organisms such as fish. Moreover, we suggest that performance dependent adaptable interactions exist in more complex networks, such as social networks.

## Supporting Information

Figure S1 **Local minima, maxima, and saddle points in the terrain.** The black circle with the letter S marks the starting position of the swarm. Local maxima are marked with a black triangle, local minima are marked with a black x, and saddle points are marked with a blue circle. A linear approximation of the separatrix, connecting the maxima and saddle points along the gradient of the terrain, is illustrated with a dotted line. A. Contour of the terrain. Recall that mountains (in red) correspond with low concentration and that valleys (in blue) correspond with high concentration. B. colored image of the terrain.
(TIF)

Figure S2
**The effect of the radii of interaction on interacting agents.** The radii of interaction control all characteristics of the group's behavior, pattern and performance. A. Median path length as a function of the radius of alignment and attraction. Strong attraction and weak alignment cause groups to attract to their centers of mass and stay in place, harming their task performance. Strong alignment and weak attraction cause excessively high conformity in the group which again, harms performance. Intermediate values around a fixed quotient of 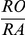 reach optimal performance. B. Alignment decreases for values lower than the fixed quotient of 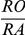. C. A weak attraction term results in high clustering by the end of the simulation almost independent of the alignment term. D. The 90th percentile path length is affected in the same manner but to a higher degree by the radii of interaction as the median path length. Simulation parameters are as in Figure 8, apart from 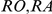.(TIF)

Figure S3
**The effect of the radii of interaction on agents with adaptable interactions.** The group movement characteristics of agents with adaptable interactions remain similar for a larger range of radii of interactions than that of interacting agents. Performance drops for values in which performance of interacting agents also drops. A. Median path length as a function of the radius of alignment and attraction. Strong attraction and weak alignment cause groups to attract to their centers of mass harming their task performance, similarly to interacting agents. As opposed to interacting agents, strong alignment and weak attraction do not harm performance. B. Alignment decreases for values lower than the fixed quotient of 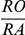, similarly to interacting agents. C. A weak attraction term results in high clustering almost independent of the alignment term, similarly to interacting agents. D. The 90th percentile path length is affected in the same manner but to a slightly higher degree by the radii of interaction as the median path length. Simulation parameters are as in Figure S2.(TIF)

Text S1 **Supporting information.** The chemical concentration map and the effect of the radii of interactions.
(DOC)

Video S1 **Movie of the simulation show independent agents moving towards the target.** Simulation parameters are as in Figure 4.
(MP4)

Video S2
**Movie of the simulation show interacting agents moving collectively towards the target.** Simulation parameters are as in Figure 4, 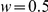.(MP4)

Video S3 **Movie of the simulation show agents with adaptable interactions moving collectively towards the target.** Simulation parameters are as in Figure 4.
(MP4)

Video S4 **Movie of the simulation shows interacting agents split and collide at the target.** Simulation parameters are as in Figure 4.
(MP4)

Video S5
**Movie of the simulation shows agents with adaptable interactions fracture.** Simulation parameters are as in Figure 4 except 
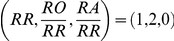
.(MP4)

Video S6
**Movie of the simulation shows agents with adaptable interactions split and collide.** Simulation parameters are as in Figure 4 except 
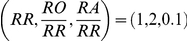
.(MP4)
