# Supplementary figures and images for: Smart Swarms of Bacteria-Inspired Agents with Performance Adaptable Interactions
Source: PLoS Comput Biol. 2011 Sep 29;7(9):e1002177. doi: 10.1371/journal.pcbi.1002177 (PMC3182867; doi:10.1371/journal.pcbi.1002177)

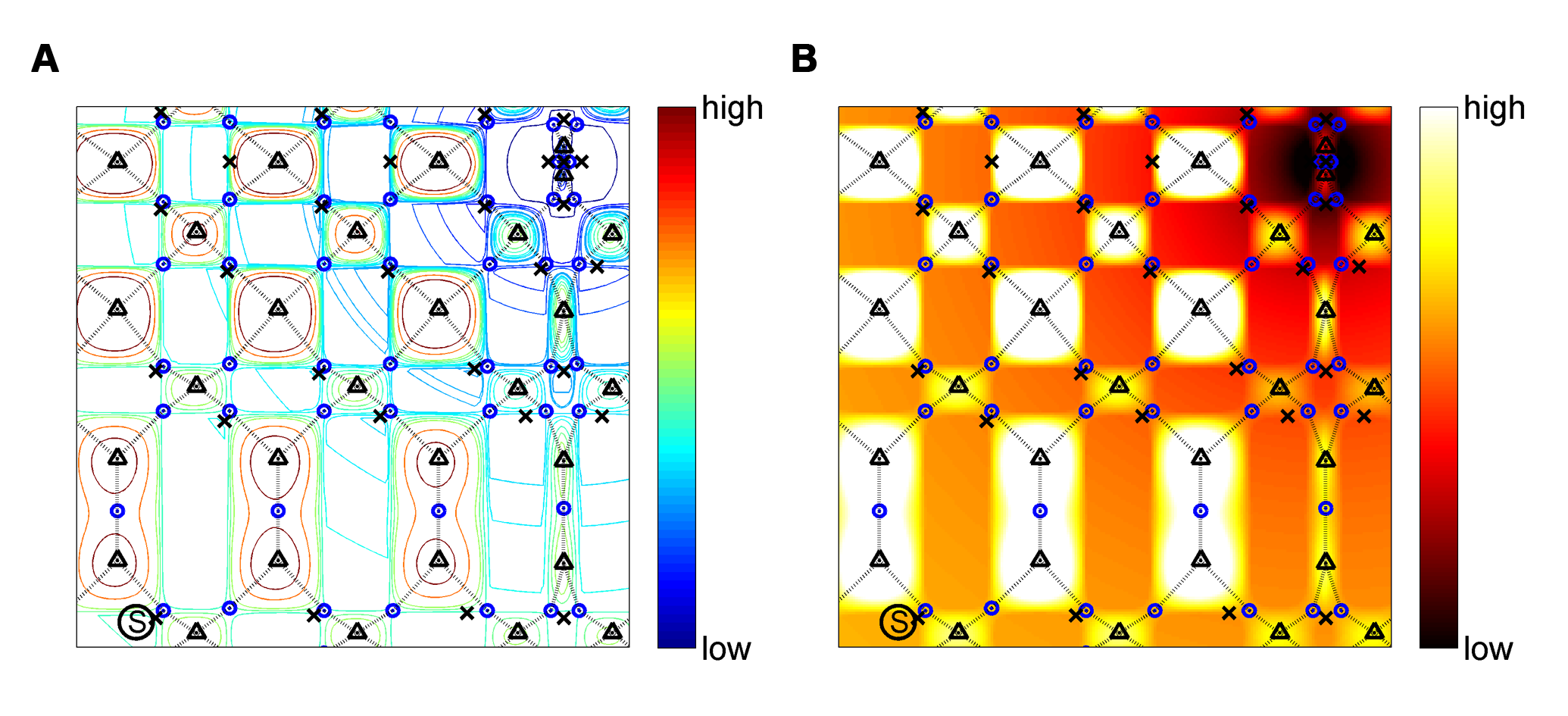

Supplement: Figure S1 — Local minima, maxima, and saddle points in the terrain. The black circle with the letter S marks the starting position of the swarm. Local maxima are marked with a black triangle, local minima are marked with a black x, and saddle points are marked with a blue circle. A linear approximation of the separatrix, connecting the maxima and saddle points along the gradient of the terrain, is illustrated with a dotted line. A. Contour of the terrain. Recall that mountains (in red) correspond with low concentration and that valleys (in blue) correspond with high concentration. B. colored image of the terrain. (TIF) [file pcbi.1002177.s001.tif]

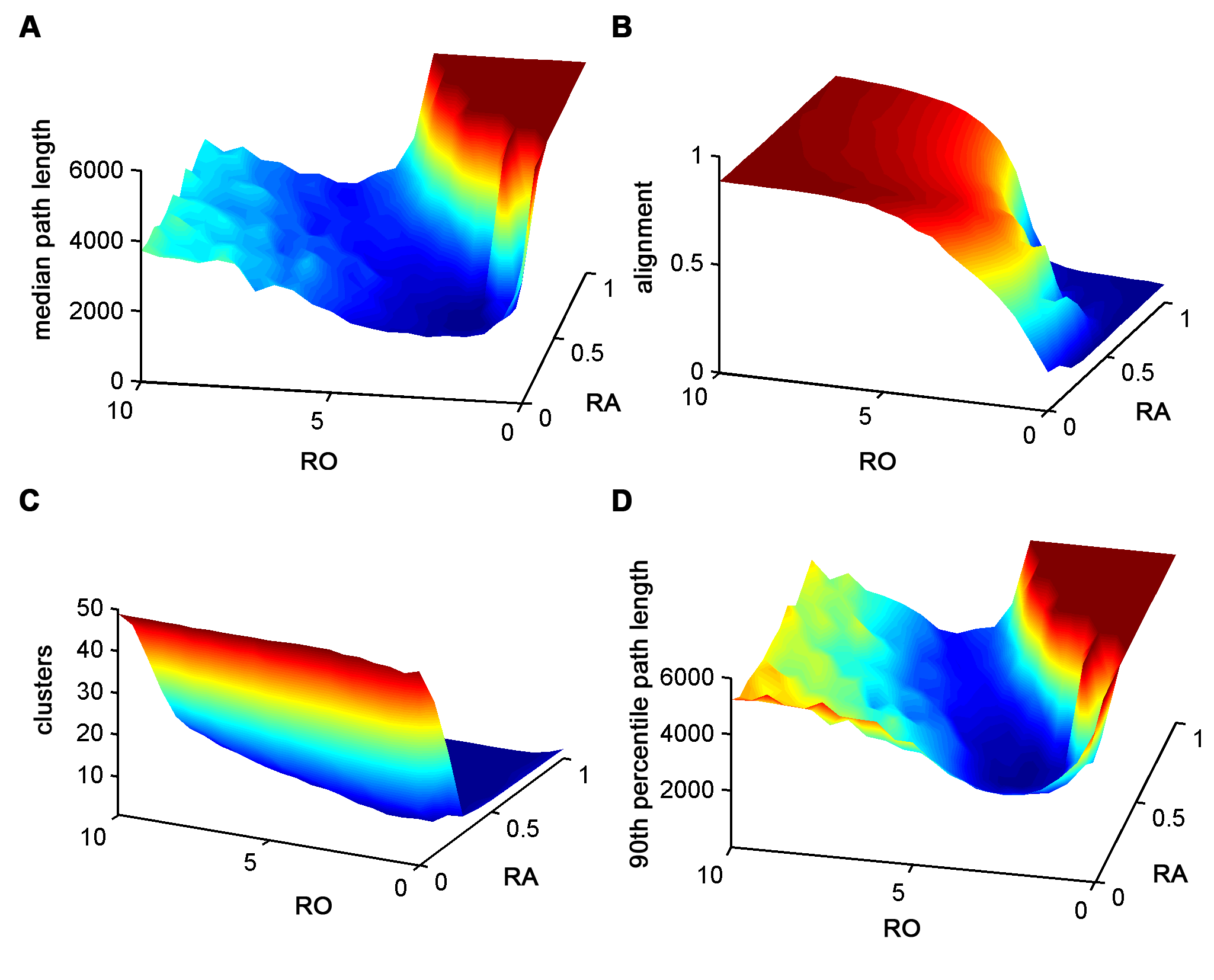

Supplement: Figure S2 — The effect of the radii of interaction on interacting agents. The radii of interaction control all characteristics of the group's behavior, pattern and performance. A. Median path length as a function of the radius of alignment and attraction. Strong attraction and weak alignment cause groups to attract to their centers of mass and stay in place, harming their task performance. Strong alignment and weak attraction cause excessively high conformity in the group which again, harms performance. Intermediate values around a fixed quotient of reach optimal performance. B. Alignment decreases for values lower than the fixed quotient of . C. A weak attraction term results in high clustering by the end of the simulation almost independent of the alignment term. D. The 90th percentile path length is affected in the same manner but to a higher degree by the radii of interaction as the median path length. Simulation parameters are as in Figure 8, apart from . (TIF) [file pcbi.1002177.s002.tif]

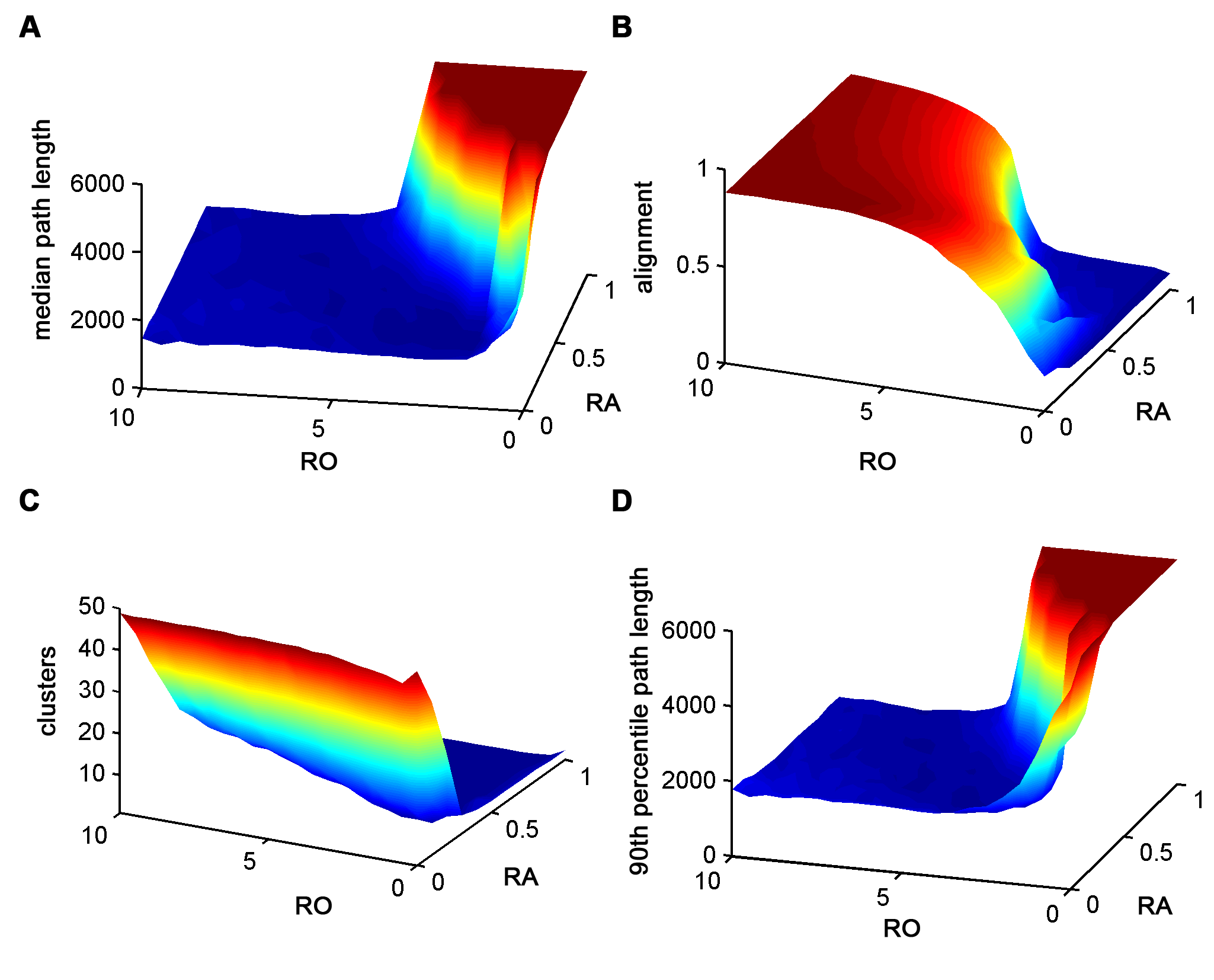

Supplement: Figure S3 — The effect of the radii of interaction on agents with adaptable interactions. The group movement characteristics of agents with adaptable interactions remain similar for a larger range of radii of interactions than that of interacting agents. Performance drops for values in which performance of interacting agents also drops. A. Median path length as a function of the radius of alignment and attraction. Strong attraction and weak alignment cause groups to attract to their centers of mass harming their task performance, similarly to interacting agents. As opposed to interacting agents, strong alignment and weak attraction do not harm performance. B. Alignment decreases for values lower than the fixed quotient of , similarly to interacting agents. C. A weak attraction term results in high clustering almost independent of the alignment term, similarly to interacting agents. D. The 90th percentile path length is affected in the same manner but to a slightly higher degree by the radii of interaction as the median path length. Simulation parameters are as in Figure S2. (TIF) [file pcbi.1002177.s003.tif]
