# Supplementary material for: Smart Swarms of Bacteria-Inspired Agents with Performance Adaptable Interactions
Source: PLoS Comput Biol. 2011 Sep 29;7(9):e1002177. doi: 10.1371/journal.pcbi.1002177 (PMC3182867; doi:10.1371/journal.pcbi.1002177)
Supplement: Text S1 — Supporting information. The chemical concentration map and the effect of the radii of interactions. (DOC) [file pcbi.1002177.s004.doc]

# Supporting Information

**The chemical concentration map**

Finding the global optimum of a function is a challenging problem due to the existence of local mimima. This becomes even more difficult when the memory and the computational resources are limited, as in the case of bacteria-inspired agents. The terrain representing the chemical concentration map in our model contained local minima, maxima, and saddle points. Figure S1 shows these points as well as a linear approximation of the separatrix, which connects the maximum and saddle points along the gradient of the terrain. Thus, this problem cannot be solved by naive gradient descent methods. Heuristic solutions, such as simulated annealing, require large jumps which are both infeasible in the case of bacteria and result in large paths. The bacteria-inspired schooling solution maintains the limited memory and computational demands and minimizes the length of the path.

**The effect of the radii of interactions**

The group's macroscopic behavior may change abruptly over a small range of values of the radii of interactions (Figure S2, see also Couzin et al. [1]). Interacting agents with strong attraction and weak alignment resulted in non-aligned groups that swarm in one place and failed to reach the target. The high attraction term drove the group to swarm around its center of mass, without synchronizing the directions of motion of the agents. For strong alignment and weak attraction, aligned groups had a large median path length due to global noise. These groups also had a high tendency to fragment because of the weak attraction. With no attraction or alignment terms, groups behaved as independent agents. They tended to fragment, did not align (if they did not fragment), and had similar path lengths as independent agents. Groups with intermediate interaction radii that maintained a constant quotient, $\frac{RO}{RA}$, between their alignment and attraction radii had intermediate to high alignment and optimal median path lengths. The 90th percentile path was affected more strongly by the radii of interactions than the median path length (Figure S2D).

Agents with adaptable interactions were less affected by the values of the radii of interactions compared to the interacting agents (Figure S3). Agents with adaptable interactions having strong attraction and weak alignment tended to swarm in a non-aligned way and had poor task performance, similar to the interacting agents. However, the performance of agents with adaptable interactions did not deteriorate due to strong alignment and weak attraction, as it had for the interacting agents. Although groups were more likely to fragment at the end of the simulation, their average alignment throughout the simulation was high and they had short path lengths. The 90th percentile path length was also high only for strong attraction and weak alignment, and was almost identical to the median path length.

## References

1. Couzin ID, Krause J, James R, Ruxton GD, Franks NR (2002) Collective Memory and Spatial Sorting in Animal Groups. J. Theor. Biol. 218: 1-11. doi:10.1006/jtbi.2002.3065

## 
